# Clinical impact and misdiagnosis of functional ophthalmological symptoms: a case report

**DOI:** 10.1192/j.eurpsy.2023.1426

**Published:** 2023-07-19

**Authors:** B. Pozuelo Moyano, K. Tzartzas, I. Kokkinakis

**Affiliations:** 1 Unisanté - Centre de médecine générale et de santé publique, Lausanne, Switzerland; 2 Département des Policliniques (DDP), Unisanté - Centre de médecine générale et de santé publique, Lausanne, Switzerland

## Abstract

**Introduction:**

The disease burden due to non-specific, functional, and somatoform disorders is high. An unknown part of these medically unexplained symptoms belongs to factitious disorders. When it comes to deciding whether a patient is able to work, it is essential to differentiate a somatoform disorder from a factitious disorder.

**Objectives:**

The aim is to highlight, on the one hand, the differences between somatoform disorder and factitious disorder and, on the other hand, the potential impact of misdiagnosis in medical expertise regarding eligibility for a disability income.

**Methods:**

A case report of Ms A. a 42-year-old Caucasian woman. She worked as a 100% fiduciary accountant until the age of 32 when she was placed on medical leave due to persistent trigeminal neuralgia. Subsequently, she developed total blindness, accompanied by distress in a crucial emotional context. A diagnosis of factitious disorder was retained by an expert psychiatrist, with severe consequences for her, such as disability income suspension and family conflict. We evaluated Ms. A. in our multidisciplinary medical expertise service for a disability income review.

**Results:**

The patient reported a total absence of light perception in both eyes (subjective), not confirmed by objective ocular examination and spécific neuro-ophthalmological examination.

Psychiatric examination revealed that Ms. A. had been sexually assaulted at age of 7 and sexually abused for a year by her teacher at age of 14. Regarding the identity of the first abuser, she describes that she *“can’t see his face”* and that the multiple sexual assaults during her teenage years took place in the classroom after school, with the teacher *“pulling down the window shades so it was totally dark.”* She explains that, defensively, to avoid thoughts related to the traumatic experience, she was heavily invested in her studies. But, at the age of 30, after separation with her first boyfriend, diffuse pain and progressively total blindness appeared.

We concluded the diagnoses of pain disorders related to psychological factors and a dissociative neurological symptom disorder with visual disturbance.

**Conclusions:**

Blindness not explained by a physiologic process may accompany trauma and psychological distress, with the search for the link between the onset of symptoms and significant unconscious psychic conflicts being crucial in the psychiatric investigation. A new diagnosis of dissociative neurological symptom disorder with visual disturbance (6B60.0) is included in the ICD-11. It is characterized by visual symptoms such as blindness, tunnel vision, diplopia, visual distortions, or hallucinations that are not consistent with a recognized disease of the nervous system, other mental, behavioral, or neurodevelopmental disorders. Differentiating this pathology from factitious disorder or simulation is essential from an insurance medicine point of view, but also for its treatment.

**Disclosure of Interest:**

None Declared

